# Significance of a Non-Thermal Plasma Treatment on LDPE Biodegradation with *Pseudomonas Aeruginosa*

**DOI:** 10.3390/ma11101925

**Published:** 2018-10-10

**Authors:** Laurence Scally, Miroslav Gulan, Lars Weigang, Patrick J. Cullen, Vladimir Milosavljevic

**Affiliations:** 1 BioPlasma Research Group, Dublin Institute of Technology, Sackville Place, Dublin 1, Dublin, Ireland; pjcullen@dit.ie; 2 School of Physical Science, Dublin City University, Dublin 8, Ireland; miroslav.gulan@gmail.com (M.G.); vm@dit.ie (V.M.); 3 Faculty of Physics, University of Belgrade, P.O.B. 368, 11000 Belgrade, Serbia; 4 School of Biological Science, Dublin City University, Glasnevin, Dublin 9, Ireland; lars.weigang2@mail.dcu.ie; 5 Department of Chemical and Environmental Engineering, University of Nottingham, Nottingham NG7 2RD, UK

**Keywords:** non-thermal plasma, biodegradation, polymers, optical emission spectroscopy, optical absorption spectroscopy, plasma treatment

## Abstract

The use of plastics has spanned across almost all aspects of day to day life. Although their uses are invaluable, they contribute to the generation of a lot of waste products that end up in the environment and end up polluting natural habitats such as forests and the ocean. By treating low-density polyethylene (LDPE) samples with non-thermal plasma in ambient air and with an addition of ≈4% CO_2_, the biodegradation of the samples can be increased due to an increase in oxidative species causing better cell adhesion and acceptance on the polymer sample surface. It was, however, found that the use of this slight addition of CO_2_ aided in the biodegradation of the LDPE samples more than with solely ambient air as the carbon bonds measured from Raman spectroscopy were seen to decrease even more with this change in gas composition and chemistry. The results show that the largest increase of polymer degradation occurs when a voltage of 32 kV is applied over 300 s and with a mixture of ambient air and CO_2_ in the ratio 25:1.

## 1. Introduction

Fossil fuels have been extensively used to fabricate various polymers that span uses from the medical to food industry and permeate multiple facets of day-to-day life. The current infrastructure of material creation allows different polymers to be fabricated through processes that give fine control of material properties and gives rise to versatile approaches to tailor these materials for multiple needs [1,2,3]. However, although they are invaluable due to their durability and ease of application to multiple areas, plastics made from fossil fuels are highly resistant to many natural processes of degradation [4]. Due to this, certain problems arise from the improper disposal of plastic waste, litter, and their long lifetime. Such issues include: (i) pollution of oceans, (ii) ingestion of plastics by animals causing contamination in the food cycle, (iii) endangerment of different species due to environmental impacts, (iv) soil contamination, and (v) introduction into water systems that feed into lines for human consumption [5,6,7,8,9,10]. The areas impacted by plastic waste and pollution will continue to suffer as the population increases and puts more demand on their generation, which ultimately puts more stress on the environment. Some of the most widely used polymers to date include low density polyethylene (LDPE), high density polyethylene (HDPE), polyethylene terephthalate (PET), polypropylene (PP), and polyvinyl chloride (PVC). These, and more, can be seen in Table 1. The percentages seen in Table 1 define how much each listed plastic contributes to the total amount of plastic pollution that currently exists. The five polymers listed (PET, LDPE, HDPE, PP, and PVC) in Table 1 contribute to a combined total of 81.5% of known plastic pollution with the remaining 18.5% coming from other plastics. Currently, the methods of plastic disposal and recycling are not able to facilitate the amount of plastic waste being created, most of which comes from plastics that have short use times (less than a year). It is reported that around 79% of plastic waste ends up in landfills or the natural environment and by 2050, there will be an estimated 12 billion tons of plastic waste existing between landfills and the natural environment [8]. From this, it is easy to see that, although plastics are well established in our daily lives and their manufacturing infrastructure is well imbedded in the industrial sector, there needs to be a serious change for a more sustainable method or alternative of plastic generation and disposal.

With the large quantity of plastics being introduced into the environment, new developments have been made to stem the quantity that remains in it by utilizing materials that have much shorter lifetimes and can still function in the place of classic plastic materials. This has led to the development and implementation of plastics and polymers that can degrade through the introduction of biological media and different environmental conditions. These are known as biodegradable polymers [11,12]. Interest in biodegradable polymers has increased in recent times to replace other synthetic polymers. Some biodegradable polymers that have come to the forefront include polylactic acid (PLA), polyglycolic acid (PGA), polyvinyl acetate (PVA), polycaprolactone (PCL), and polymers with fibrous blends that consist of biomaterials such as starch [13]. Methods that can be used to fabricate biodegradable polymers include the use of microorganism growths and plant matter extracts [13]. As of late, more methods have been developed in order to create biopolymers and create polymer blends in order to achieve better results for applications such as medical implantation, tissue growth, replacements for other plastic fiber resins, and food packaging [14,15]. Although the use of biodegradable polymers will help to eliminate many negative aftereffects of fossil fuel-based polymer waste, research is still needed to fully understand and optimize their generation for specific uses to better advance various applications.

Published works have shown that the current-state-of-art plasma systems can be used to aid in the abatement and destruction of volatile organic compounds (VOCs) as well as aiding in the degradation of polymeric materials. The use of packed bed non-thermal plasma (NTP) reactors, as well as pre-treatment of VOCs before introduction to a biotrickling filtration (BTF) stage, has shown that the removal of 95%+ of VOCs can be achieved [28,29,30]. The use of packed bed NTP reactors has been shown to be of use to increase the efficiency of plasma discharge as the introduction of ferroelectric materials (BaTIO_3_, NaNO_2_, TiO_4_) aids in the generation of a stronger electric field via polarization. Furthermore, this gives rise to the formation of higher energy electrons within the NTP discharge region. From this, a higher rate of energy transfer can arise and form more reaction pathways for VOC breakdown [29]. From these reaction mechanisms, the VOCs may dissociate into smaller constituent parts that are less harmful. These smaller constituent parts of VOCs can then be filtered through zeolite screening or BTF in order to further increase the system’s VOC degradation efficiency [29]. The use of BTF post-NTP treatment allows biodegradation to occur. This helps in the further breakdown of VOCs and also helps to trap and degrade harmful compounds formed from the breakdown of the VOCs after NTP treatment [29,30,31,32]. Some of the VOCs that these systems have been shown to help degrade include styrene, toluene, benzene, PLA, and polyolefins. Current work strongly indicates that the use of BTF systems are a step forward in purification processes and that they can be further improved upon by introducing an NTP treatment stage [28,29,30,31,32,33,34].

Being able to modify and functionalize biodegradable polymers to tailor specific properties and characteristics is a very important step in the research of alternative material selections to increase and improve the quality of more natural and greener products. This also extends to knowing how to best tackle the existing pollution to try and decrease it and to potentially create a modification process to functionalize polymers, such as PET, PP, and LDPE, to reduce their lifetimes in landfills and oceans. Current processes that are already installed to produce classically non-biodegradable polymers may be difficult to change quickly, and so an alternative may be needed that can be implemented into these industrial manufacturing processes to achieve a reduction in waste production while maintaining high quality of the produced materials for their intended uses. One such method to do this may be to implement installations of non-thermal plasma (NTP) systems. It has been shown that the colonization of polymer surfaces by microorganisms depends on the functional groups present on the polymer surface, but it is also generally accepted that samples with higher hydrophilicity may give rise to an easier colonization process for these microorganisms [17]. For LDPE and HDPE, it has been found that oxidized groups on the sample surface are easier for microorganisms to degrade and that the adhesion of microorganisms can be increased by creating a more oxidized and hydrophilic surface [16,17,35,36,37].

## 2. Results and Discussion

Characterizing the NTP treatment system was carried out by implementing optical diagnostics as described in Section 3.4. By utilizing the non-intrusive nature of optical measurements, the gas chemistry along the profile of the sample treatment area was determined for the use of ambient air and ambient air with a CO_2_ admixture at a ratio of 25:1 (ambient air:CO_2_). By using the optical emission spectroscopy (OES) and optical absorption spectroscopy (OAS) results from the optical measurement and comparing them with the changes seen in the Raman spectra, the optimum parameter setting to induce the greatest amount of biodegradation of LDPE can be ascertained.

### 2.1. Optical Diagnostics

From the measurements of the plasma discharge at each power setting and with the use of solely ambient air, as well as with the introduction of CO_2_, there were many common emission species and ozone was detected very clearly. However, the use of just ambient air led to the formation of excited atomic nitrogen (N I), and with CO_2_ as an admixture with ambient air, the generation of C_2_ was detected. The formation of these reactive species, and the others detected in this work, could be inferred through the use of the electron energy distribution function (EEDF) obtained from the line ratio of (N_2_-337/N_2_^+^-391) [38]. From this, the most likely paths for reaction mechanisms could be highlighted and the energetics that occur at the sample’s surface during treatment give rise to a better understanding of possible surface modifications.

**Figure 1:**
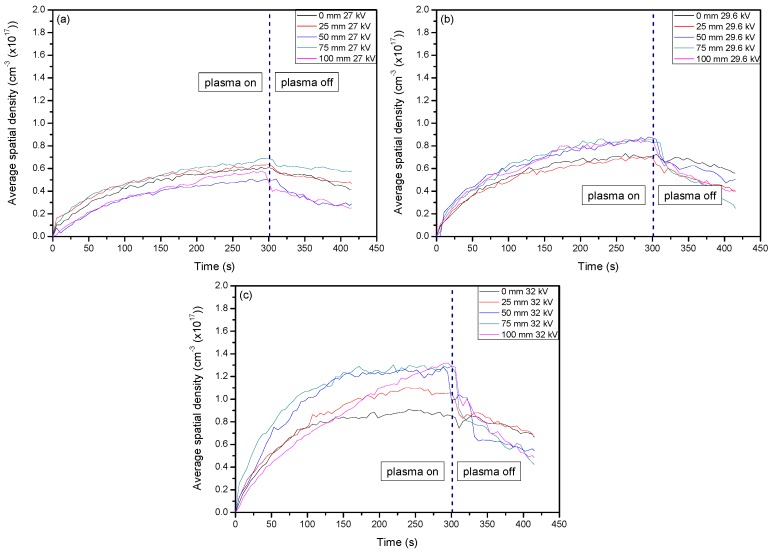
Average spatial density profile of O_3_ at 253.7 nm when ambient air was the only gas present for plasma discharge. The line divides values at the point when the plasma system was set to stop generating plasma. (**a**–**c**) show the changes of O_3_ average spatial density with respect to voltage.

The first set of results to be compared are the average spatial densities of O_3_ that were measured throughout the treatment area as well as temporally to show the evolution of this powerful oxidant. Figure 1a–c shows the spatial and temporal evolution of ozone for the three applied voltages and demonstrates the potential to have an amount of it remain to interact with the sample surface even after plasma generation has been stopped. From each graph of Figure 1, it can be seen that at the very beginning, the average density of O_3_ increases relatively the same for each setting. However, when a higher voltage was applied, there was a larger difference between each set of values and the values from measurements that were more central in the system have higher maximum values compared to the edge areas. This was due to species such as O_2_, OH, and H_2_O having longer residence times within the applied electrical field, which allowed them to aid in more reaction mechanisms that create O_3_ as the energetics were larger here due to their residence time in the direct electrical field. The possible reaction mechanisms can be seen below in Equations (1)–(3), where *M* is a third body atom or molecule such as O, N_2_*, or OH [38,39,40].

(1) $$O_{2}+e_{fast}\;\to •O+•O+\;e_{slow}\;$$

(2) $$•O+{\;H}_{2}O+\;↔2\mathrm{OH}\;$$

(3) $$•O+{\;O}_{2}+\;M^{*}\to •O_{3}+\;M$$

**Figure 2:**
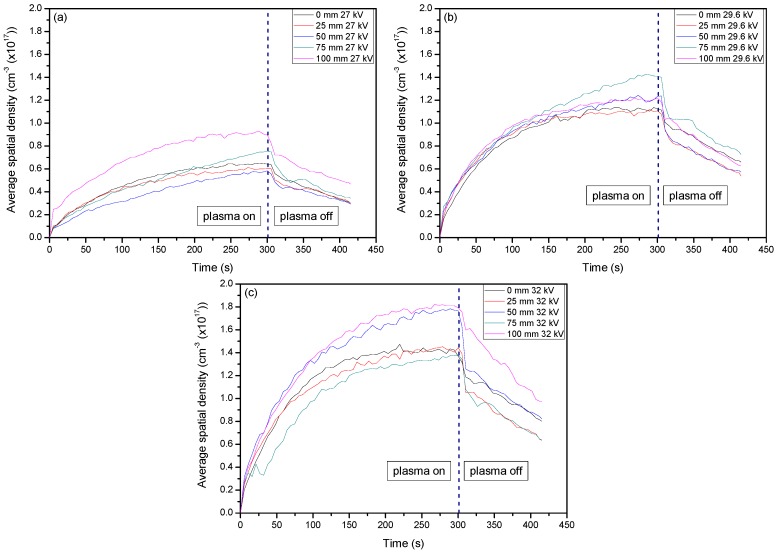
Average spatial density profile of O_3_ when CO_2_ was introduced to the plasma discharge. The line divides values at the point when the plasma system was set to stop generating plasma. (**a**–**c**) show the changes of O_3_ average spatial density with respect to the change of voltage settings.

Figure 2a–c shows the average spatial density of O_3_ when CO_2_ was introduced as a slight admixture into the ambient air being used. By having a CO_2_ additive in the plasma discharge, the formation of O_3_ was higher than when solely ambient air is used. Setting the flow rate of ambient air to 1 L min^−1^ with the introduction of CO_2_ being set at 0.04 L min^−1^ impacted the creation of O_3_ through Reactions (4) and (5) that could then go on to aid in Reaction (3) and the formation of O_3_ [40]. From these results, it can already be seen that the tailoring of the plasma system to generate more of a certain reactive species is possible. From the optical absorption spectroscopy (OAS) results, it can be seen that the formation of a highly oxidative species was maximized by simply introducing a slight amount of an additive gas, CO_2_ in this case. The formation of O_3_ was important for this study as it could help to form a more polar sample surface during plasma treatment, and as previously stated, it is generally taken that the more polar a sample surface, the better cell adhesion will be. For this study, and indeed for many other applications, this is an important note to take into consideration when forming new technologies and methods for sample processing. Optical diagnostics are also of great importance to form a better understanding of the gas chemistry that is induced. From here, the optical emission spectroscopy (OES), optical absorption spectroscopy (OAS), and Raman spectroscopy must be looked at to form a more concrete conclusion as to what gas chemistry is most desirable for polymer degradation in this study.

(4) $${\mathrm{CO}}_{2}+e_{fast}\;\to \mathrm{CO}\left(a^{\prime 3}\Sigma \right)\;+•O+\;e_{slow}$$

(5) $${\mathrm{CO}}_{2}+e_{fast}\;\to \mathrm{CO}\left(A^{2}\Pi \right)\;+•O+\;e_{slow}$$

**Figure 3:**
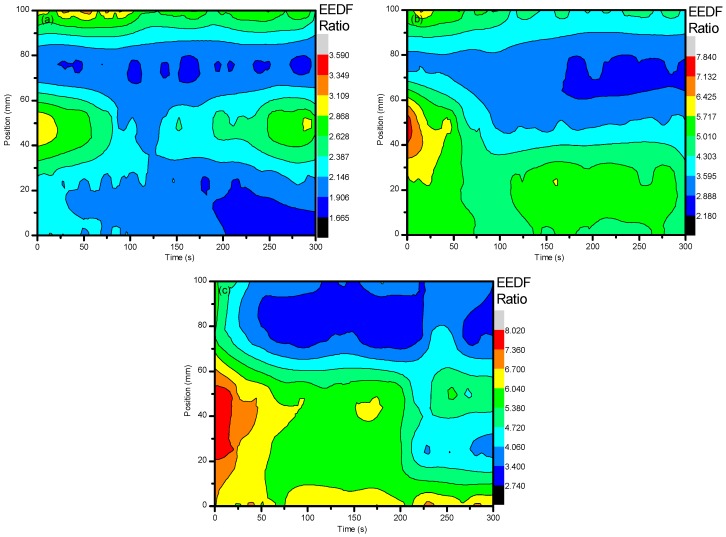
The line ratio of (N_2_-337/N_2_^+^-391) to give the EEDF when using ambient air. This portrays how the electron energies were altered with the different parameter settings, most importantly the variation of working gas composition. (**a**–**c**) represent discharge at 27, 29.6, and 32 kV, respectively.

The EEDF results from the use of ambient air in the central regions (25–75 mm) show that, over time, the electron energies began to dissipate while the edges of the system (0 mm and 100 mm) maintained their values. This can be described as the same reasoning for the formation of O_3_ over time. Due to the increased residence time within the system, the excited nitrogen species became saturated and underwent a temporal evolution of excitation and deexcitation processes, while the edges of the system had more consistent populations of ground state N_2_ to interact with that did not alter the spatial evolution as much. From Figure 3, it can then be seen that the electron energies were distributed more towards the low energy with increases in voltage. These changes in electron energetics are very important to understand the formation of other species within the plasma and how they alter the sample for degradation purposes.

**Figure 4:**
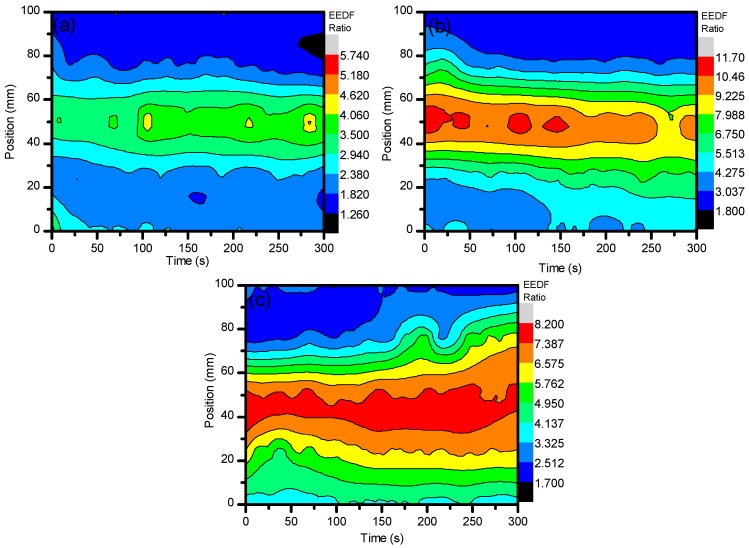
Shows the line ratio of (N_2_-337/N_2_^+^-391) when using ambient air with ≈3.8% CO_2_. This portrays how the electron energies were altered with the different parameter settings, most importantly the variation of working gas composition. (**a**–**c**) represent discharge at 27, 29.6, and 32 kV, respectively.

With an addition of CO_2_ at ≈3.8% to the system, a drastic change in the electron energies was incurred. By using the same line ratio method and spectral line profiles (N_2_-337/N_2_^+^-391) that were used for the EEDF of ambient air, a comparison could be easily drawn between the two experimental setups and easily diagnose the energetics of the use of the different gases. When CO_2_ was introduced to the system, the EEDF was much more inclined to be sensitive to the impact of low energy electrons. As can be seen, the values for the EEDF were much higher when CO_2_ was introduced, even as a small percentage of the total working gas used. However, there was a greater spread of energetics for the air: CO_2_ mixture compared to that of just ambient air. As can be seen, the spread of values for ambient air when 27 kV, 29.6 kV, and 32 kV were applied was 1.93, 5.66, and 5.28, respectively. As CO_2_ was introduced, the spread became 4.48, 9.90, and 6.50 for 27 kV, 29.6 kV, and 32 kV, respectively. This fluctuation can be seen in Figure 4a–c below and occurred with a distribution of higher values from the center of the system out to the edges, showing that lower energetics were detected at this point. This was due the quenching mechanisms of N_2_* by CO_2_. From Equations (3)–(5), it can be seen that energetic electrons dissociate CO_2_ into CO and O, which aid in the formation of O_3_. Since we saw a much higher generation of O_3_ with the introduction of CO_2_, it is good to assume that CO_2_ was dissociated through electron impact. Given that the excitation cross-section and electronegativity of CO_2_ is higher than that of N_2_, any energetic electrons that were generated were more likely to interact with CO_2_ over N_2_, causing an indirect quenching of N_2_^+^. From this, it can be understood how the electron energetics were kept at a relatively low level as they did not have the necessary time to undergo more interactions with the applied electric field as they imparted their energies for dissociative mechanisms.

**Figure 5:**
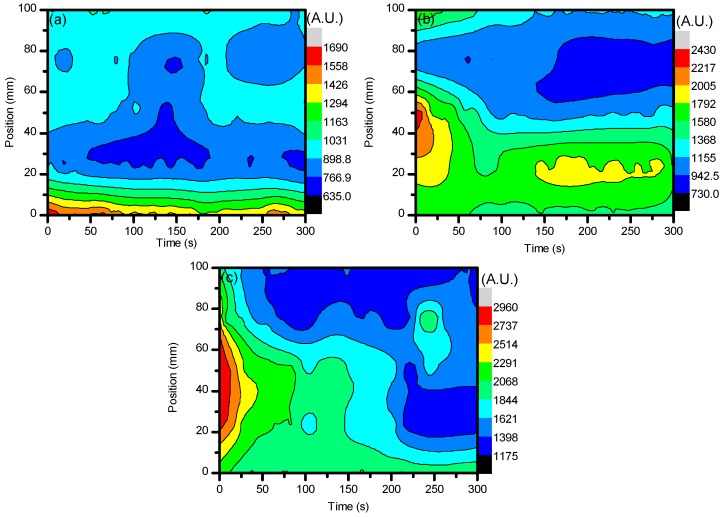
The above shows the spatial and temporal evolution of OH when using ambient air as the sole working gas in the plasma system. (**a**–**c**) represent 27 kV, 29.6 kV, and 32 kV, respectively.

**Figure 6:**
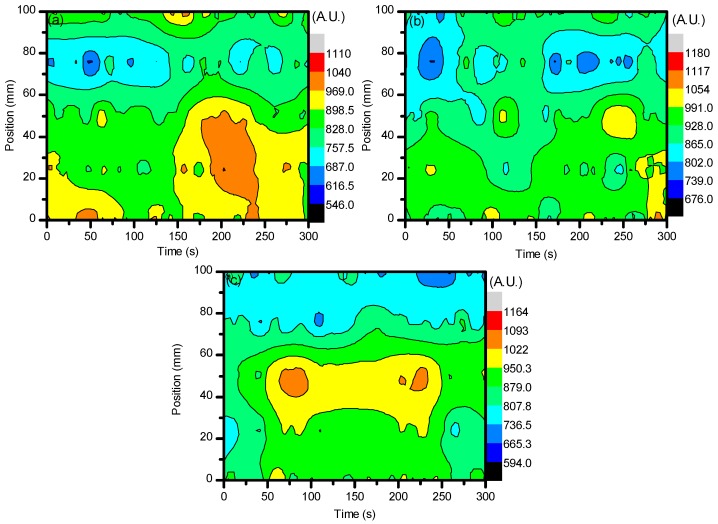
The evolution of O I with respect to the spatial and temporal profile of the ambient air plasma discharge is shown here with an applied voltage of 27 kV, 29.6 kV, and 32 kV, shown in (**a**–**c**) respectively.

Knowing that there were oxidative species being generated within the system is important, as it was suspected that these may aid in the adhesion and biodegradation of the LDPE samples being treated. For this, there was a focus on reactive species containing oxygen, and through the use of OES, atomic oxygen (O I) was found at 777 nm and OH was found at 309 nm. Figure 5a–c shows the generation of OH with the use of ambient air for the different applied voltages. It can be seen that an appreciable amount of OH was generated throughout the system, but there are points that show higher generation over others. As the voltage increased, the generation of OH became more prevalent throughout the entirety of the system, but there was still a decrease in OH emissions over time, which indicates that there was a saturation event that blocked the formation of any new OH. From what we found with the generation of O_3_ and from what can be seen of O I in Figure 6a–c, there may have been a reduction in the production of OH due to recombination effects of OH back to H_2_O, which allowed more O I to become available over time, as described in Equation (2). This can be seen as the fluctuation of O I seen in Figure 6a–c would have represented changes in available pathways and over time, this would have been influenced by O_3_ dissociation and the recombination of 2OH back into H_2_O + O.

**Figure 7:**
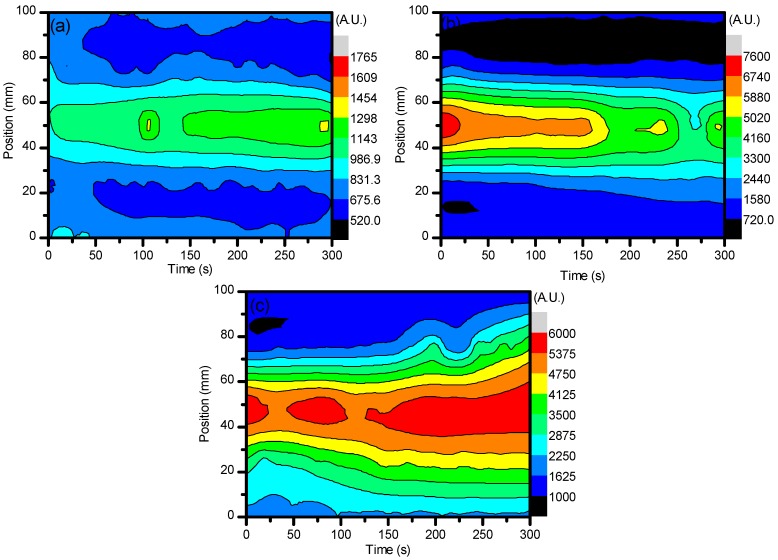
The formation of OH as detected by OES when the ambient air introduced to the system contained ≈3.8% CO_2_. (**a**–**c**) represent voltages 27 kV, 29.6 kV, and 32 kV, respectively.

**Figure 8:**
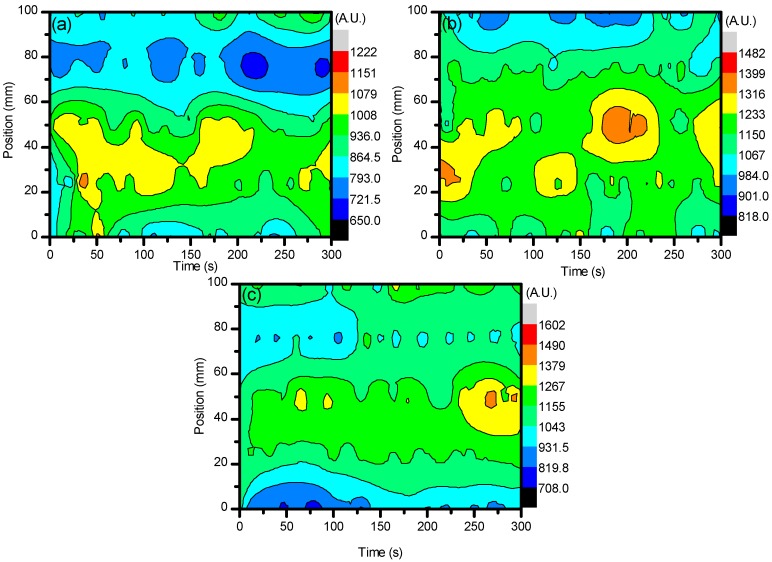
The formation of O I when the ambient air introduced to the system contained ≈3.8% CO_2_. (**a**–**c**) represent the applied voltages of 27 kV, 29.6 kV, and 32 kV respectively.

The generation of OH and O I when CO_2_ was introduced to the system during plasma discharge both increased appreciably. This can be seen in Figure 7a–c and Figure 8a–c below. From the previous results of the EEDF line ratio in Figure 4a–c, it can be seen that the electron energies were more distributed throughout the lower energy portion for the plasma being generated when CO_2_ was introduced, and as previously stated, this was due to the dissociation of CO_2_ into CO + O from more appropriately energized electrons through collisional processes that decreases the potential energy that the electrons can reach. This aided in a higher formation of O I species, which in turn took part in the formation of OH and O_3_. However, there was a much larger increase of OH compared to the increase of O I due to recombination mechanisms that transformed CO back into CO_2_, as well as the continuous increase of O_3_ that was present at much higher levels compared to those measured with solely ambient air.

### 2.2. Weight Loss and Raman Spectroscopy

**Figure 9:**
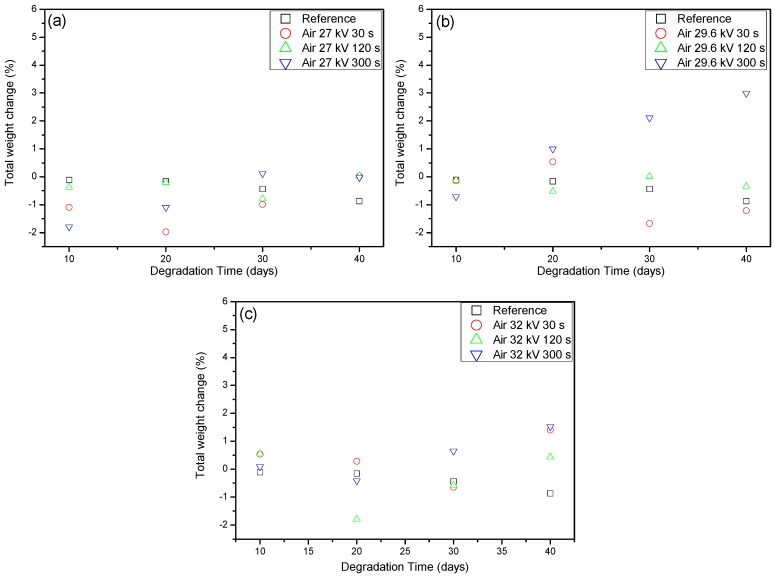
Percentage weight change of LDPE when introduced to the bacterial broth of *Ps. aeruginosa* for 10, 20, 30, and 40 days. These represent the percentage weight change in LDPE after degradation with plasma treatment times of 30, 120, and 300 s (**a**) shows changes for the different times at 27 kV (**b**) shows changes for the different times at 29.6 kV (**c**) shows changes for the different times at 32 kV.

**Figure 10:**
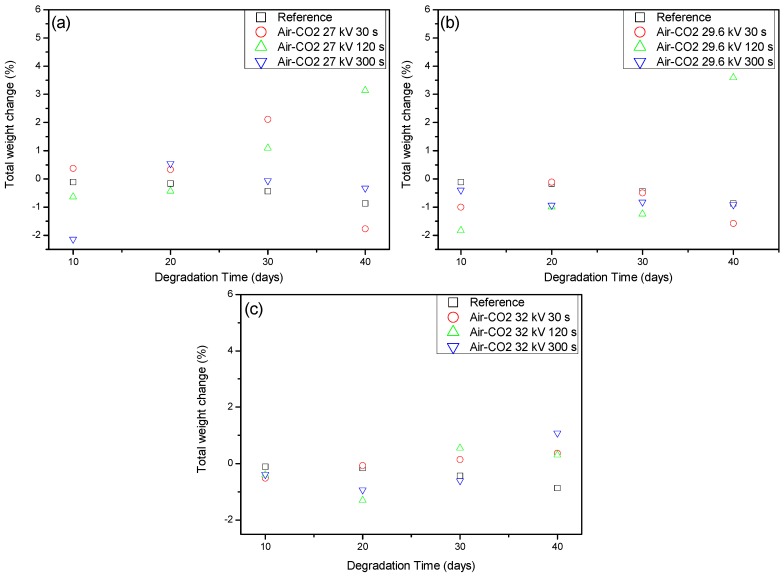
Percentage weight change of LDPE when introduced to the bacterial broth of *Ps. aeruginosa* for 10, 20, 30, and 40 days after being treated with plasma discharge containing ≈4% CO_2_ in ambient air. These represent the percentage weight change in LDPE after degradation with plasma treatment times of 30, 120, and 300 s (**a**) shows changes for the different times at 27 kV (**b**) shows changes for the different times at 29.6 kV (**c**) shows changes for the different times at 32 kV

From the results obtained from the OES and OAS measurements, it can be seen that the introduction of CO_2_, even at small quantities, aided in the formation of reactive species. These oxidative species were specifically targeted as they can help to functionalize a sample’s surface to become more polar, and therefore more accepting of cell adhesion for various cultures. This was thought to support the biodegradation of the treated samples as the cells should more readily bind to the sample surface and begin decomposition of the carbon bonds that make up the polymer. In order to determine the amount of degradation that the treated samples underwent, the samples were weighed after plasma treatment and after introduction to the bacteria *Ps. aeruginosa.* The results of the weight loss method for degradation detection can be seen in Figure 9a–c and Figure 10a–c. As can be seen from these results, the only sample that consistently lost weight was the reference sample, which was sterilized and untreated LDPE. The other samples showed sporadic losses and gains in weight with no discernible pattern or trend. There is a possibility that the samples that were treated could gain more weight over the degradation time, but this was difficult to ascertain without more data on this. From this, it could not be discerned whether there was any degradation present or not. So, in order to determine what was occurring and whether or not the introduction of the bacteria after plasma treatment had any significant impact, Raman spectroscopy measurements were carried out to monitor any changes in the bonds of the samples. Most importantly, it was of great interest to measure any changes in the carbon bonds of the sample to show that the samples were being degraded from the bacteria using carbon as an energy source for growth and proliferation.

**Figure 11:**
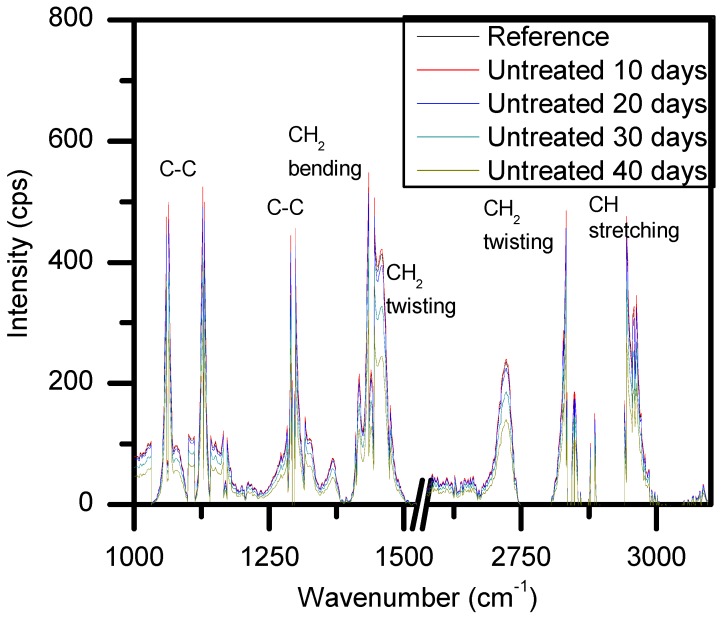
Representation of the typical Raman spectra of LDPE and the affiliated carbon bonds to help determine degradation of the samples.

Shown in Figure 11 is a reference sample of LDPE that was sterilized but not introduced to the bacterial broth for degradation, as well as degraded reference samples that were introduced to the bacterial broth medium without any plasma treatment. This shows the bonds that were of interest for our study and how they were important to measure in order to determine the changes in the carbon groups of the polymer as a function of degradation time. If there were decreases seen from these measurements, then this could be taken as a better sign of biodegradation compared to the weighing of the samples. Figure 11 sets the baseline for how much degradation may occur without any extra treatments and sets the standard for degradation after plasma treatment and whether or not it could be increased.

**Figure 12:**
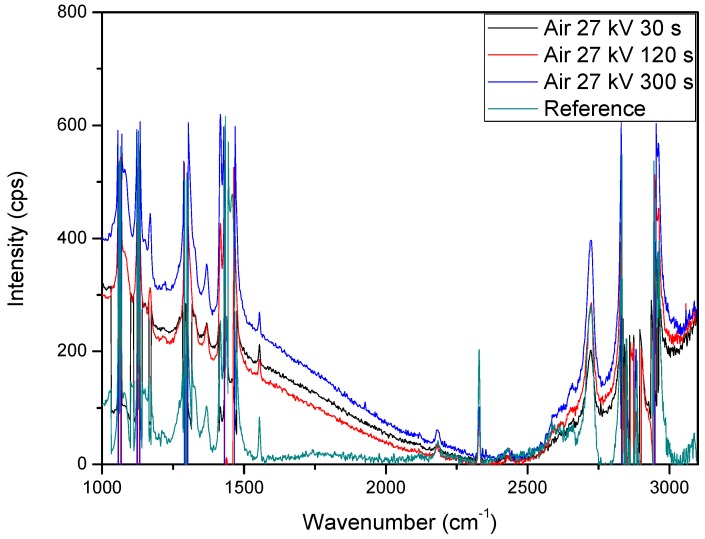
Comparison of treated samples to an untreated sample that have undergone 10 days of degradation to show the presence of fluorescence due to organic matter adhesion on the sample surface after plasma treatment with ambient air.

After taking the measurements of all samples with the Raman spectrometer, the reason as to why there were sporadic changes in the weight of treated samples was found. When carrying out the Raman measurements, there was a noticeable difference in the spectra. At either end, there was a large curve that continued to skew the results. It was more dominant from 2250 cm^−1^ to 1000 cm^−1^ and can be seen in Figure 12 with the untreated sample added as a comparison. The reason for this deviation is fluorescence. Fluorescence can occur for many reasons and may be associated with a contaminated sample, but it can also be due to biological matter being on a sample. Given the use of bacterial cells for degradation of the LDPE samples, and with their handling being the same as every other sample, it is safe to assume that the fluorescence seen in their spectra is due to the presence of organic matter that was a residual of the bacterial cells. By taking this into account, it can be seen why the results from weighing the samples was so sporadic and created an unreliable method to determine the degradation of the samples. Given this, it could be determined that there was much better cell adhesion on the sample surface, which agreed with previous assumptions and validates the use of plasma to optimize the grafting of bacterial cells to the LDPE samples as a step towards optimized biodegradation. The comparison of fluorescence seen in treated samples compared to the reference sample can be seen in Figure 12.

**Figure 13:**
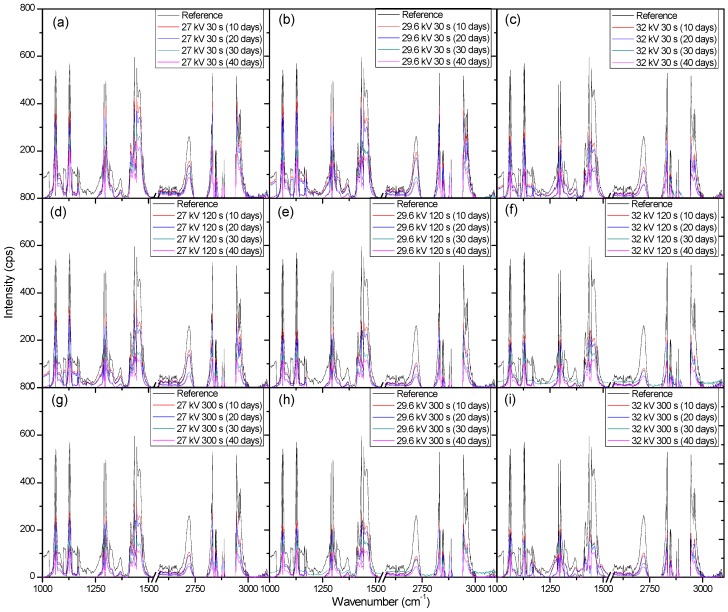
Raman spectroscopy measurements of plasma treated LDPE after their introduction into the bacterial broth to obtain biodegradation. The operating gas for plasma discharge was ambient air. (**a**–**i**) show how exposure time and applied voltage during plasma treatment impact the degradation process of LDPE. (**a**–**c**), (**d**–**f**), and (**g**–**i**) show how the changes in voltage impact the degradation of LDPE while maintaining a constant treatment time. (**a**,**d**,**g**), (**b**,**e**,**h**), and (**c**,**f**,**i**) show how the changes in treatment time impact the degradation of LDPE while maintaining a constant applied voltage.

**Figure 14:**
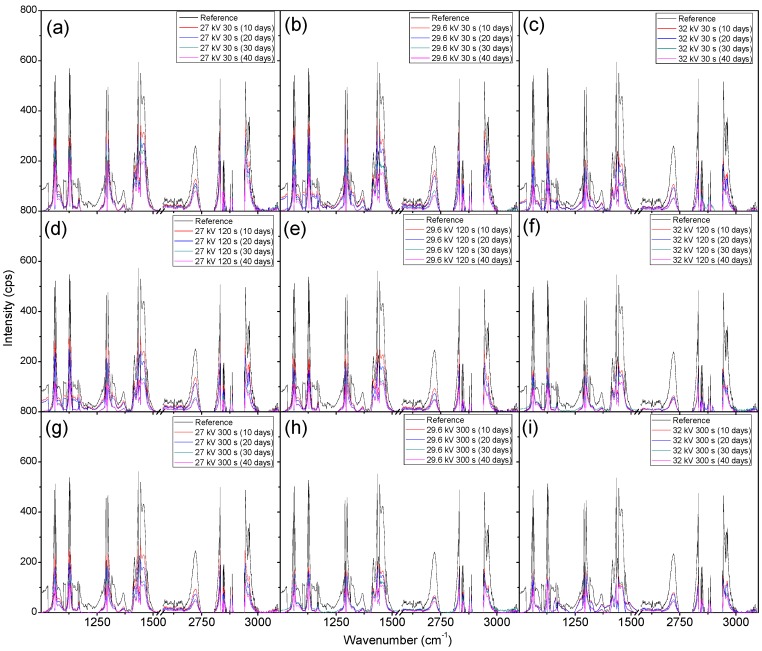
Raman spectroscopy measurements of LDPE after their introduction into the bacterial broth to obtain biodegradation to determine the impact ≈4% CO_2_ had in the plasma discharge. (**a**–**i**) show how exposure time and applied voltage during plasma treatment impact the degradation process of LDPE. (**a**–**c**), (**d**–**f**), and (**g**–**i**) show how the changes in voltage impact the degradation of LDPE while maintaining a constant treatment time. (**a**,**d**,**g**), (**b**,**e**,**h**), and (**c**,**f**,**i**) show how the changes in treatment time impact the degradation of LDPE while maintaining a constant applied voltage.

After taking into consideration the presence of fluorescence in the treated LDPE samples to explain the weight gains seen in Figure 9 and Figure 10, the ability to discern the degradation of the polymer samples comes down to the measurement of the carbon bonds highlighted in Figure 11. It can be seen that there was an issue that occurred from the measured fluorescence, and that is an offset of each peak that needed correction to properly identify the changes that may have arisen from degradation within the bacterial broth. A polynomial fit was applied to each spectra in order to subtract the best fit baseline in order to analyze the peaks’ presence for any changes that they underwent. The full set of Raman measurements for ambient air plasma discharge can be seen in Figure 13, and the impact on biodegradation when ≈3.8% CO_2_ was introduced can be seen in Figure 14.

From the results obtained with Raman spectroscopy, it can be seen that there was degradation occurring for each sample. This shows that, even though the weighing of the samples proved ineffective at determining the degradation of the LDPE samples due to the excess bacterial cells adhered onto the polymer surface, the use of Raman spectroscopy highlighted the decrease in the carbon bonds of the polymer strips. This shows that with plasma treatment, there was a mixture of good cell grafting onto the samples’ surfaces without negatively impacting the degradation of the samples. From Figure 13, the change in treatment time allowed for higher biodegradation, as does an increase in voltage. However, there was also a beneficial impact on the biodegradation of LDPE when treating the polymer with a working gas containing CO_2_, as the samples treated with ≈3.8% CO_2_ in ambient air showed slightly more biodegradation compared to the samples treated with solely ambient air. Interestingly, it seems that there was a selectivity in which carbon bonds the bacteria broke down. This is put forward as the peaks associated with carbon bonds did not seem to decrease at the same rate. There was some proportionality to these changes, but the bacteria may have broken down and processed more loosely bound and weaker bonds first, which could have given rise to a slightly uneven degradation process across the sample.

## 3. Methods and Materials

### 3.1. Non-Thermal Plasma Treatment

**Figure 15:**
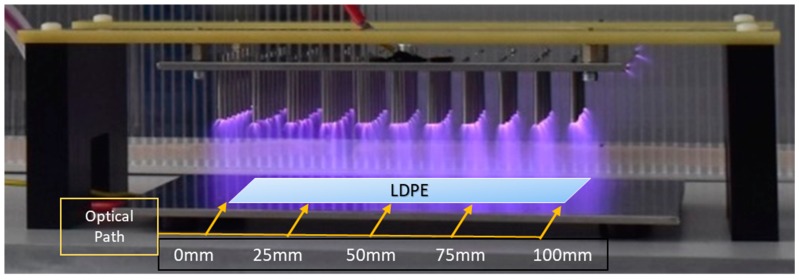
The NTP pin system that was used. Not seen is the plastic box that was used to cover the system during treatments and optical measurements. LDPE samples were placed within the plasma discharge for the duration of their treatment. Shown is plasma discharge in ambient air.

The use of a novel dielectric barrier discharge (DBD) NTP system was employed to treat the samples of LDPE. This DBD system utilizes a newly developed pin design that allows for the generation of NTP with an AC power supply without the use of any insulating material placed between the electrodes as a dielectric material. Although no solid material was used (plastic sheet, insulating cover, etc.), the gases used would act as a dielectric to an extent to hinder electrical arcing from one plate to another before plasma discharge could occur. The system itself uses two steel plates as electrodes, the ground electrode being flat and the high voltage electrode having an array of optimally placed pins. The pins on the high voltage electrode were initially tested with them all being placed so their points would sit on the same plane, but this produced a non-homogenous discharge and generally created plasma along the edge of the system rather than the full way through. To optimize this, the pins were then arranged in a convex pattern that had the central pins down closer to the ground electrode with the distance from pin to ground plate decreasing slightly as they were placed further from the center. This caused plasma discharge to occur throughout the entire system as the applied electric field was not focused along the edge points of the high voltage electrode and was spread out more homogeneously between the electrodes. For this experiment, the distance from one plate to another was 10 cm, but from the central pin tip to the ground electrode, it was 7 cm. The system can be seen in Figure 15. The power supply was able to generate plasma discharge with a resonant frequency of 52 kHz and voltages of 27, 29.6, and 32 kV. The duty cycle was kept at 118 μs with a discharge frequency of 1 kHz. The power at these parameter settings were 3.74, 5.66, and 7.67 W for 27, 29.6, and 32 kV, respectively.

Covering the system was a plastic container that had holes bored into it to allow for optical studies, gas input, contain reactive species and reduce loss through diffusion, to allow ambient air to pervade through the system, and to allow excess build-up of gas during the introduction of the ambient air:CO_2_ mixture to escape so as not to cause any unequal distribution of the gases. The LDPE samples were placed on the ground electrode of the system after it was cleaned with ethanol and allowed to dry in order to sterilize the surface and decrease the risk of contamination of the samples. The samples were treated on both sides, so for the treatment of 30 s, the sample was treated on both sides for 30 s to try to modify the total surface area of the LDPE strips. When using the gas mixture of ambient air and CO_2_, the gas was given time to fill up the container so as to make sure there was an equal gas distribution throughout the system. After plasma treatment on both sides, the samples were left inside the container to allow any post-discharge species to interact with and modify them (i.e., O_3_).

### 3.2. LDPE Sterilization and Bacterial Broth

The use of LDPE for this experiment was to try and determine the impact that NTP treatment has on the biodegradation of classically non-biodegradable polymers. However, it has been found that some bacteria may degrade certain polymers by using the polymer as a carbon source for consumption to gain energy for cell growth. However, even before treating the LDPE samples, they needed to be prepped. Sheets of LDPE were washed with a mix of 30% deionized water and 70% ethanol [41,42]. After this wash, they were oven dried at 50 °C. After they were successfully dried, they were then brought to the NTP system for treatment. They were placed into the system after the ground electrode was cleaned with 100% ethanol and air dried. The sterilized sheets were then treated for 30, 120, and 300 s at voltages of 27. 29.6, and 32 kV in ambient air and a 25:1 mix of ambient air to CO_2_. The treatments consisted of treating the sterilized samples on both sides to ensure total surface area interaction with the plasma discharge. After being treated, the samples were cut into 1 × 5 cm strips and placed into a broth media that contained the bacteria *Ps. aeruginosa* for incubation for periods of 10, 20, 30, and 40 days to investigate the effect of biodegradation. After incubation, the plastic strips were placed in a 10 mL solution of 0.9% NaCl for 2 h and then vortexed for 10 min. This was to remove the biofilm layer and measure the density of it, which was found to be between 1.1 × 10^6^ and 1.5 × 10^6^ CFU. Slight fluctuations were found to occur, but this was assumed to be due to remnants of the cell growth remaining on the samples even after washing, as shown in Figure 12. The growth medium, a nutrient basal media, was also tested to ascertain the concentration of cells within it. The growth medium concentration was consistently found to be 2.7 × 10^7^ CFU.

Cultivating the bacteria *Ps. aeruginosa* was done according to work carried out by Kyaw et al. [41]. The broth media that was made to incubate the bacteria and samples was made from the following: 12.5 g/L K_2_HPO_4_, 43.8 g/L KH_2_PO_4_, 1.0 g/L (NH_4_)SO_4_, 0.1 g/L MgSO_4_·7H_2_O with 5 mL of trace elements solution: 0.232 g/L H_3_BO_3_, 0.174 g/L ZnSO_4_·7H_2_O, 0.116 g/L FeSO_4_(NH_4_)2SO_4_·6H_2_O, 0.096 g/L CoSO_4_·7H_2_O, 0.022 g/L ((NH_4_)_6_Mo_7_)_24_·4H_2_O, 0.008 g/L CuSO_4_·5H_2_O, and 0.008 g/L MnSO_4_·4H_2_O. Inoculation and incubation was carried out under sterile conditions. A total of 30 mL of nutrient basal media was added to a falcon tube. After this, 10 strips of LDPE were added to the tube. A total of 0.6 mL of bacterium in 0.85% saline solution were added to tube; the initial concentration for each incubation was kept at 0.5 McFarland Standard. Incubation was maintained at 37 °C and tubes were placed in a rotary shaker at 120 rpm. The tests were performed in triplicate and the tubes were regularly tested for cell growth and made sure that there was no contamination present. When the incubation of the LDPE strip in the media was over, they were removed and washed with a 2% sodium dodecyl solution (SDS) for 4 h and dried. After this, they were placed back into tubes containing deionized water and washed in a sonic bath for 30 min to remove any excess residue that may have been present. When this was finished, the strips were then dried overnight in an oven at 50 °C. Once dried, the samples were placed on a weighing scales to determine if there was any measurable loss after degradation in the bacterial broth. This gravimetric method did not give the results that were expected as there was no consistent pattern or trend in the values obtained for the weights of each batch of samples. The results that were obtained showed very small loss values and also showed higher weights compared to the values they gave before introduction to the bacterial broth. This was found to be due to fluctuations in organic matter being adhered to the sample surface as is shown in Figure 12.

### 3.3. Optical Emission and Absorption

The OES and OAS both used an Edmund Optics CCD spectrometer (Edmund Optics Inc., Barrington, NJ, USA) that has a wavelength dependent resolution of 0.6–1.8 nm. Because of this, some of the peaks that were measured were in fact an amalgamation of multiple species. An example of this is can be seen at 777 nm, which was in fact an overlapping of three peaks that could not be fully resolved with the spectrometer used. These three emission peaks would be from O I with λ = 777.194, 777.417, and 777.539 nm and transitions of 3_S_^5^$S_{2}^{o}$–3_p_^5^P_1,2,3_, respectively, and an upper energy level of 10.47 eV [43]. For this experiment, however, the total intensity measured at 777 nm was sufficient as this included all O I emission lines of interest. When taking the OES measurements of the plasma discharge, the acquisition time was 1.2 s with a delay of 3.8 s and a total of 61 acquisitions. This was to cover the fill 300 s of plasma discharge that was used for the maximum treatment time of the LDPE samples. These measurements were carried out over three voltages (27 kV, 29.6 kV, and 32 kV) for ambient air and ambient air:CO_2_ at a 25:1 mixture ratio at five different points along the treatment area of the sample. The points of measurement were at 0, 25, 50, 75, and 100 mm from the left most section of the plasma discharge to the right most side.

OAS measurements were taken along the same positions as the OES measurements and also used the same acquisition time and delay settings, but were measured for a period of 415 s to gain information on the changes in O_3_ post-discharge. Overall there were 84 spectra recorded for the OAS measurement of each parameter setting. Although the same spectrometer as was used in the OES measurements, a UV/Vis/NIR light source was used as a reference to detect changes in light intensity and thus determine the average spatial density of O_3_. The light source used was a BDS130 deuterium/tungsten lamp (B&W Tek, Newark, DE, USA) with a spectral output of 190–2500 nm. The optical path used when recording the OAS data was 29 cm. After recording the data from the OAS experiment, Equation (6) was used to determine the average spatial density of O_3_, where *D*(*t*) is the density (cm^−3^), *L* is the optical path (cm), *I*(0) is the reference intensity with no plasma discharge, *I*(*t*) is the measured intensity during and after plasma discharge, and *σ*(*λ*) is the wavelength dependent absorption cross-section for the species of interest. For O_3_ at 253.7 nm, the absorption cross-section is 1.154 × 10^−17^ cm^2^. For OAS, the power supply timer was set to 300 s so it would automatically shut off while the measurements were still being taken in order to show the changes in O_3_ over time during post-discharge.

(6) $$D\left(t\right)=\;\frac{1}{\sigma \left(\lambda \right)\;L}ln\frac{I\left(0\right)}{I\left(t\right)}$$

### 3.4. Raman Spectroscopy

In order to determine the changes in carbon bonds to detect signs of biodegradation, Raman spectroscopy was implemented. In order to carry out these measurements, the DXR SmartRaman Spectrometer from Thermo Fisher Scientific Ltd (Waltham, MA, USA) was used. The chemical structure of LDPE consists of C–C, CH_2_, CH–CH_2_, and CH_2_–CH_2_ bonds. Throughout the structure, there are no polar groups, and since the change in carbon bonds is of interest, Raman spectroscopy is suitable as the bonds that are sought after tend to have strong signals compared to infrared measurement. A 780 nm diode laser at 120 mW was used to carry out the measurements and detect the chemical groups within the polymer. The setup utilized a CCD and a universal sampling accessory with a 50 μm slit aperture. The measurements that each sample underwent consisted of 10 exposures with each exposure lasting 15 s. This was done at three random sampling points for each sample to obtain a better averaging of the measured spectra for analysis. Once the spectra were obtained, it was necessary to process the data using a polynomial fit baseline as there was a high amount of fluorescence that distorted the peaks of the spectra. This baseline procedure was carried out in Origin Pro 8^TM^ (8, OriginLab, Northampton, MA, USA). After this baseline procedure was carried out, an averaging of the data from the three random sampling points was carried out for each sample and then plotted.

## 4. Conclusions

It has been found that the use of NTP discharge for the treatment of LDPE strips leads to an increase of its biodegradation in a bacterial broth media containing *Ps. aeruginosa*. The premise of this experiment was to try to optimize a treatment procedure in which biodegradation can be increased. It is well known that NTP discharges in ambient air can generate multiple oxygen containing particles and that these can alter the surface of a sample to have it become more polar. From this, the addition of a slight amount of CO_2_ would increase the amount of oxygen containing particles to further the amount of polar functional groups that may form on a treated samples surface. From the results obtained via OES and OAS, this was found to be the case with an increase in O I, OH, and O_3_ being obtained when the ambient air contained ≈4% CO_2_. From this, and comparing the results from the Raman measurements, it was found that this increase in oxidative species in the discharge and polar groups on the sample surface leads to an increase in biodegradation compared to an untreated sample for all treated samples. However, the introduction of CO_2_ increased the biodegradation further compared to solely ambient air plasma discharge treated samples which highlights the importance of optical diagnostics in determining the gas chemistry to optimize the application of NTP systems to various areas.

## Figures and Tables

**Table 1:** Information to compare plastic polymers that are resistant to biodegradation and those that are more readily able to degrade through natural means [13,14,15,16,17,18,19,20,21,22,23,24,25,26,27].

Polymer	Uses	Structure	Contribution to Plastic Pollution %	Means of Degradation
*Low/no biodegradability*				
PET	Clothing fibers, food and liquid containers, engineering resins.	[C_10_H_9_O_4_]_n_	12.8	UV exposure, thermal oxidation, *Ideonella sakaiensis*.
LDPE	Lab equipment, plastic bags, food packaging.	[C_2_H_4_]_n_	23.9	UV exposure, oxidising solvents, *Lysinibacillus xylanilyticus*, *Pseudomonas*, and *Aspergillus niger*
HDPE	Plastic bottles, food containers, corrosion protectors, 3-D printing filament.	[C_2_H_4_]_n_	17.6	UV exposure, oxidative solvents, hydrolysis.
PP	Dielectric sheets, medical implantations, piping systems, hinges.	[C_3_H_6_]_n_	24.3	UV exposure, microbial communities mixed with starch.
PVC	Electrical cables, flooring, window insulation.	[C_2_H_3_Cl]_n_	2.9	UV exposure, *Phanerochaete chrysosporium*, *Lentinus tigrinus*, *Aspergillus niger*, *Aspergillus sydowi*
*Biodegradable*				
PLA	Medical implants, packaging material, injection molding.	[C_3_H_4_O_2_]_n_		*Amycolatopsis* and *Saccharotrix*.
PGA	Medical suture, food packaging, tissue engineering.	[C_2_H_2_O_2_]_n_		Hydrolysis.
PVA	Wood glue, nonwoven binder, primer, adhesive.	[C_4_H_6_O_2_]_n_		Filamentous fungi, bacterial, fungal species, algae.
PCL	Tissue repair scaffold, targeted drug delivery, dentistry, herbicide containers.	[C_6_H_10_O_2_]_n_		*Penicillium* and *Aspergillus*.
